# Explainable AI and Reinforcement Learning—A Systematic Review of Current Approaches and Trends

**DOI:** 10.3389/frai.2021.550030

**Published:** 2021-05-20

**Authors:** Lindsay Wells, Tomasz Bednarz

**Affiliations:** ^1^Expanded Perception and Interaction Center, Faculty of Art and Design, University of New South Wales, Sydney, NSW, Australia; ^2^Data61, Commonwealth Scientific and Industrial Research Organisation, Sydney, NSW, Australia

**Keywords:** explainable AI, reinforcement learning, artificial intelligence, visualization, machine learning

## Abstract

Research into Explainable Artificial Intelligence (XAI) has been increasing in recent years as a response to the need for increased transparency and trust in AI. This is particularly important as AI is used in sensitive domains with societal, ethical, and safety implications. Work in XAI has primarily focused on Machine Learning (ML) for classification, decision, or action, with detailed systematic reviews already undertaken. This review looks to explore current approaches and limitations for XAI in the area of Reinforcement Learning (RL). From 520 search results, 25 studies (including 5 snowball sampled) are reviewed, highlighting visualization, query-based explanations, policy summarization, human-in-the-loop collaboration, and verification as trends in this area. Limitations in the studies are presented, particularly a lack of user studies, and the prevalence of toy-examples and difficulties providing understandable explanations. Areas for future study are identified, including immersive visualization, and symbolic representation.

## Introduction

*Explainable Artificial Intelligence* (XAI) is a growing area of research and is quickly becoming one of the more pertinent sub-topics of Artificial Intelligence (AI). AI systems are being used in increasingly sensitive domains with potentially large-scale social, ethical, and safety implications, with systems for autonomous driving, weather simulations, medical diagnosis, behavior recognition, digital twins, facial recognition, business optimization, and security just to name a few. With this increased sensitivity and increased ubiquity comes inevitable questions of trust, bias, accountability, and process—i.e., how did the machine come to a certain conclusion? (Glass et al., 2008). These concerns arise from the fact that, generally, the most popular and potentially most powerful part of AI—Machine Learning (ML)—is essentially a *black-box*, with data input into a trained neural network, which then outputs a classification, decision, or action. The inner workings of these algorithms are a complete mystery to the lay-person (usually the person interacting with the AI). The algorithms can even be difficult for data scientists to understand or interpret. While the architecture and mathematics involved are well-defined, very little is known about how to interpret (let alone explain), the inner state of the neural network. Interaction with such systems are fraught with disuse (failure to rely on reliable automation), and misuse (over reliance on unreliable automation) (Pynadath et al., 2018).

This black-box scenario makes it difficult for end-users to trust the system they are interacting with. When an AI system produces an unexpected output, this lack of trust often results in skepticism and possibly even rejection on the part of the end-user. It is not clear if the result is “correct” or as a result of some flaw or bias in the creation of the AI system that led to the model being overfit on training data not representative of wide range of examples in the real world, or underfit, not sufficiently modeling the complexities of the target environment. These errors may have considerable side effects, such as unsafe resultant behaviors in factories due to misclassification, unfair treatment of members of society, unlawful actions, or financial impact on companies employing AI solutions. Marcus and Davis (2019) describe a number of these issues in their book *Rebooting AI*. They argue that current approaches to AI are not “on a path to get us to AI that is safe, smart, or reliable” (p. 23).

XAI research in the context of Machine Learning and deep learning aims to look inside this black-box and extract information or explanations as to why the algorithm came to the conclusion or action that it did. In addition to providing tools to assist with trust and accountability, XAI assists with debugging and bias in Machine Learning. The inputs and outputs and network design of Machine Learning algorithms are ultimately still decided with human input (human-in-the loop), and as such are often subject to human errors or bias. Explanations from XAI enabled algorithms may uncover potential flaws or issues with this design (e.g., are certain completely irrelevant features in the input image becoming too much of a factor in outputs?). XAI aims to tackle these problems, providing the end-user with increased confidence, and increased trust in the machine. Recent reviews into XAI have already been conducted, with the most recent being Biran and Cotton (2017), and Miller et al. (2017). These reviews focus on data-driven Machine Learning explanations. Recently Anjomshoae et al. (2019) published a systematic literature review on goal-driven explainable AI, which encompassed Reinforcement Learning (RL), although the review did not provide any specific commentary on approaches used within that area. These reviews indicate that XAI is a growing area of importance, and this is also reflected in a recent move by Google to release a range of XAI tools.^1^ Furthering the need for research in the area of XAI is the recent General Data Protection Regulation in the EU, which has a provision for the right to explanations (Carey, 2018).

In the broader ML space, the review of 23 articles by Miller et al. (2017) determined that human behavioral experiments were rare. Anjomshoae et al. (2019) reviewed 62 papers and found that after text-style explanations, which were present in 47% of papers, explanations in the form of visualization were the next most common, seen in 21% of papers. Visualization presents a dynamic and exploratory way of finding meaning from the ML black-box algorithms. A considerable amount of work has already gone into the concept of “saliency maps” which highlight areas of the input image that were of importance to the outcome, see Adebayo et al. (2018).

Following on from these previous reviews, the current work aims to examine XAI within the scope of *RL*. RL agents generally leverage a *Markov Decision Process (MDP)*, whereby at each timestep, an *action* is selected given a certain input set of observations (*state*), to maximize a given *reward*. During compute runs, the agent learns which actions result in higher rewards (factoring in a *discount factor* for obtaining long-term rewards, such as winning the game) through a carefully moderated process of exploration and exploitation. Popularly, RL has been used successfully by the *DeepMind* team to produce agents capable of better than human-level performance in complex games like *GO* (Silver et al., 2016), and a suite of Atari games (Mnih et al., 2015).

In the next section, we will qualify the reasoning for selecting RL as an area for further investigation in terms of XAI and describe the guiding research questions of this work. Then, the methodology used for the systematic literature review will be described, and the results of the review will be presented.

## Background

This work investigates RL specifically due to the unique challenges and potential benefits of XAI applied to the RL space. The concept of XAI even in agent-based AI system has been considered as early as 1994, in work by Johnson (1994) who described an approach for querying an intelligent agent and generating explanations. The system was domain-independent, implemented for a simulated fighter-pilot agent. The agent itself did not use for its approach, however there are several similarities to current RL work, as the theory behind how an explanation should be worded or generated remains the same. The agent was able to explain a decision made by going back to that point in time and “repeatedly and systematically” (p. 32) modifying the situation state, and observing the different actions the agent would take in order to form a mapping between states and actions.

### Benefits

As mentioned above, XAI aims to combat the issues of trust and confidence in AI, a topic which is particularly important when safety is a major factor. Applications such as autonomous vehicles or robotics where the robot takes in observations of the environment around it and performs actions where the result could have an impact on safety are an area where trust and accountability are pertinent (Araiza-Illan and Eder, 2019). Determining why a robot took the action it did (and by extension knowing what factors it considered) in a human-understandable way plays a big part of building a trust that the robot is indeed making intelligent and safe decisions. This could even lead to building a rapport with the robot, making working with it more efficient as their behaviors may become more predictable. Diagnosing what went wrong when a robot or autonomous car is involved in an incident would also benefit from XAI, where we could query the machine about why it took actions in the lead up to the incident, which would allow designers to not only prevent further incidents, but help with accountability or possible insurance or ethical claims (e.g., was the autonomous car at fault, was there a fault in the decision making of the car, or was another third party at fault?).

Another benefit is that RL agents often learn behaviors which are unique and can identify new strategies or policies previously not thought of. A recent example of this was a game of hide-and-seek where agents learned strategies to exploit the physics system of the game to overcome what was intended by the developers to be walls that could not be passed (Baker et al., 2019). Extracting from the black box how these strategies were learned, or under what circumstances these strategies were learned could result in useful new knowledge for decision making or optimization. As Stamper and Moore (2019) point out, analysis of agents playing the Atari 2600 game *Space Invaders* exhibited similar decision-making behaviors to expert human players (e.g., keeping the invaders in a square formation, and destroying right-most enemies first to slow down the rate of advancement), however in other games investigated, the strategies varied more from human play. Understanding and articulating these strategies may result in new knowledge on how to optimally play these games, but also enhance recommendation systems for informed decision making. A quote by Zhuang et al. (2017) sums up the current situation well: “*[…] people and computers can both play chess, it is far from clear whether they do it the same way*.”

### Challenges

A challenge in providing XAI for RL is that it usually involves a large number of decisions made over a period of time, often aiming to provide the next action at real-time speeds. Compared to standard ML techniques where decisions can happen in isolation or are unrelated to each other, RL explanations generally will need to encompass a set of actions that were related in some way (e.g., outputting explanations such as “I did actions A,B,C to avoid a penalty for Z”).

Another challenge is the fact that RL agents are generally trained without using training data (with the exception of where human-replay data is used, such as in Vinyals et al., 2017), and instead learning is facilitated by a feedback loop (observations) from performing actions within an environment. This makes it challenging to generate human-readable explanations. While the observation and action spaces may be labeled in sensible ways, having no human-labeled training data linking actions and observations makes it challenging to produce valid explanations.

Further adding to the difficulties in XAI, is that developing an AI system that is explainable and transparent can be at odds with companies that have explicit commercial interests which they may not want exposed by overly verbose AI. It can also raise issues around protecting their IP, maintaining a competitive advantage, and the additional costs involved with implementing XAI (Mohanty and Vyas, 2018).

## Methodology and Research Questions

With XAI becoming increasingly important for a range of reasons previously described, and work in this area beginning to grow, it is important to take stock of the current approaches in order to find similarities, themes, and avenues for further research. As such, the guiding research questions for this review are:

RQ1: What approaches exist for producing explainable output for Reinforcement Learning?

RQ2: What are the limitations of studies in the area of XAI for Reinforcement Learning?

It is worth taking a moment to clarify the meaning of “explanation” and “explainability” in this paper. In the case of a systematic literature review using these words as search terms, search results will appear for a multitude of meanings and interpretations of these words. For example, “explainability” might refer to something which makes a system more transparent or understandable. An “explanation” may refer to something which describes the actions, decisions, or beliefs of an AI system. “Explainablity” however may also refer to logging or verifications, or an AI system that can be queried or visualized. During the filtering process described in the next section, no restrictions were placed on how the authors defined or interpreted these terms.

Given these research questions, the following section describes the methodology for searching the extant literature for information to address them.

## Selection of Literature

To examine the current state of the literature, a systematic literature review using a methodology adapted from Kitchenham et al. (2009) was performed. Searches were conducted on the ACM, IEEExplorer, Science Direct, and Springer Link digital libraries, using Boolean search queries, taking the term “Reinforcement Learning” and combining it with the terms “data visualization,” “information visualization,” “explanation,” “explainable,” “explainable ai,” “XAI,” “black box,” “visual analytics,” “hybrid analytics,” and “human in the loop.” The full set of search term combinations can be found in Supplementary Materials.

In addition, papers were filtered using the following criteria:

- *recent paper*: papers had to be published within the last 5 years (i.e., since 2014 at time of writing);
- *relevancy*: papers had to be relevant to the topic of *RL* (papers which spoke about general agent-based AI system or RL from a human psychology perspective were excluded) and *explainability* (i.e., papers which did not describe an approach for explaining the actions or policy of an agent were excluded);
- *accessibility*: papers needed to be accessible *via* the portals previously described;
- *singularity*: duplicate papers were excluded; and
- *full paper*: extended abstracts and other short papers were excluded.

**Figure 1:**
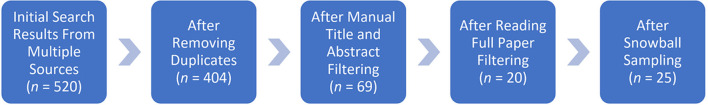
Number of papers included in review after various stages of filtering.

As Figure 1 illustrates, a total of 520 papers were gathered, which was reduced to 404 after filtering out duplicate results using the EndNote software “Find Duplicates” feature. The titles and abstracts of these papers were reviewed for relevance to the domain of RL and XAI, of which 69 were deemed relevant using the relevancy measure described above. These papers were then read fully to determine relevance to the domain. The remaining 20 papers after this stage of filtering constitute the main analysis of this paper.

The jump down from 69 to 20 may seem surprising, however due to the search terms, a number of papers mentioned RL in the body for purposes of describing AI systems generally for the reader, or in some cases RL was used as the technique for generating explanations for a different form of AI such as classification. Such use of the term “Reinforcement Learning” could not be determined until the full paper was examined. Many filtered papers advertised frameworks or implementations for XAI in ML in general and were picked up by the search terms for RL as the papers described the broad spectrum of Machine Learning which encompasses RL. However, these papers ultimately just described typical classification problems instead.

In addition, 5 papers were added to the review, using a snowball sampling technique (Greenhalgh and Peacock, 2005), where if a relevant sounding paper was cited by a reviewed paper, it was subsequently assessed, and if deemed relevant added to the pool of papers for review (15 papers were examined during this stage).

Before going into detail of some of the approaches for XAI in RL, the following section explores at a high level the core themes in the 25 papers reviewed in terms of domain and scope, in order to paint a picture of the current state of the research space.

## Summary of Literature

Selected papers were categorized and analyzed based upon four main topics: domain, publication type, year, and purpose. A full summary table of the selected papers and information about each is provided in Supplementary Materials.

### Domain

**Figure 2:**
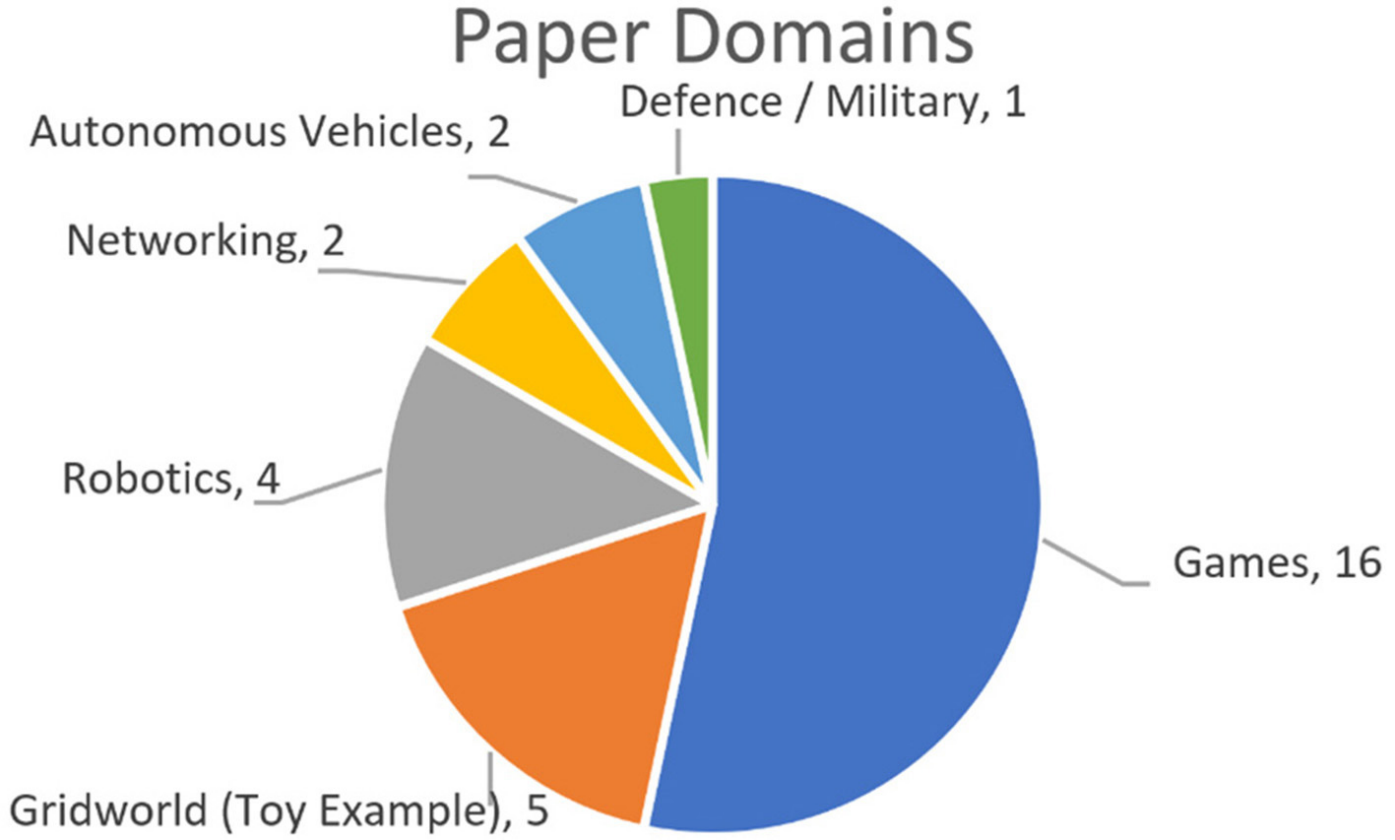
Categorization of papers by domain. Note that some papers were in multiple domains.

Papers were categorized based upon the featured subject domain(s) they focused on (either in their implementation, or theoretical domain). It was possible for each paper to be in multiple categories. The distribution of papers across the categories is summarized in Figure 2, and expanded upon in this section.

**Figure 3:**
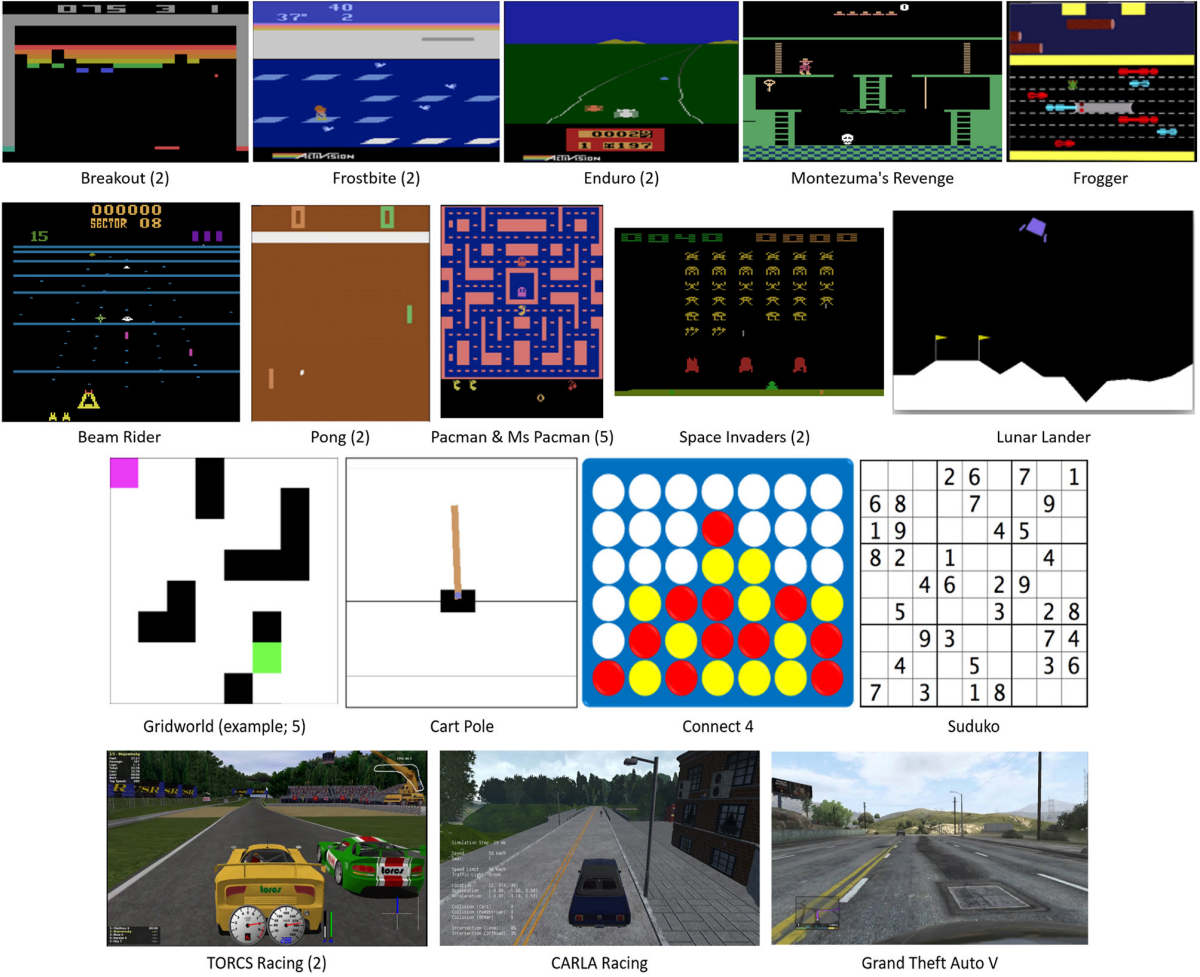
Screenshots of the game-based applications in the studied papers. Where more than one paper used that game, the number of papers using the game are shown in brackets.

The majority of papers (16; 64.0%) focused their examples on the domain of video games (particularly Atari games, given recent popularity due to DeepMind's success), however choice of target game was generally quite broad spread, with the only game utilized in more than one paper was Pac-Man, as illustrated in Figure 3. Most common after this were examples using a basic grid-world environment with a navigation task (5 papers), and examples in robotics (4 papers).

The domain of networking tasks such as video bitrate monitoring and cloud-based applications appeared in 2 papers. An area that was expected to have greater representation was autonomous vehicles (and this is validated by the mention of this area of RL frequently in the reviewed papers), however this area was the focus of only 2 papers.

Finally, one paper was written from a defense/military perspective. It should be noted that only 6 papers attempted to apply their implementation to multiple example situations, however even in these cases, it was from within the same domain (e.g., multiple types of games).

### Publication Type

The primary outlet for the reviewed papers was conference proceedings (16 papers), with only 3 papers published in journals. Another 4 papers were from the open access repository arXiv,^2^ 3 of which were found as part of the snowball sampling process described previously. One publication (Pynadath et al., 2018) was a book chapter published in “Human and Machine Learning.”

### Year

**Figure 4:**
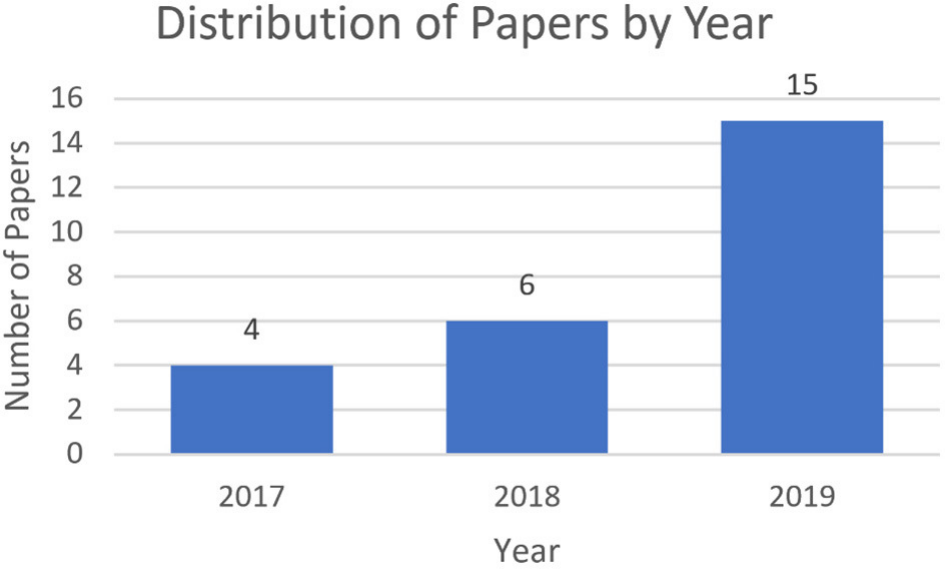
Distribution of surveyed papers by year, indicating an increase of academic interest in this area.

The majority of papers found were published in 2019 (15 papers), while only 6 were published in 2018, and 4 in 2017 (see Figure 4). This indicates that research into attempting produce explainable RL agents is an area of considerable growth. As we will see, given the sudden increase in publications, there is a reasonable amount of cross-over between some streams of research, and ideally these researchers may consolidate their work and progress together, rather than in parallel, into the future.

### Purpose/Scope

The reviewed papers presented a mixture of papers attempting to establish a theory or model (6 papers), while others primarily focused on introducing a new method for explainable RL (18 papers).

The primary purpose or focus of the reviewed papers was coded down to 5 core topics as shown in 5 (it was possible for a paper to be assigned to multiple topics): *human collaboration* (7 papers); *visualization* (9 papers); *policy summarization* (10 papers); *query-based explanations* (5 papers); and *verification* (1 paper). This distribution of purposes is consistent with the findings in the Anjomshoae et al. (2019) review, which found a high number of visualization-based explanation systems.

**Figure 5:**
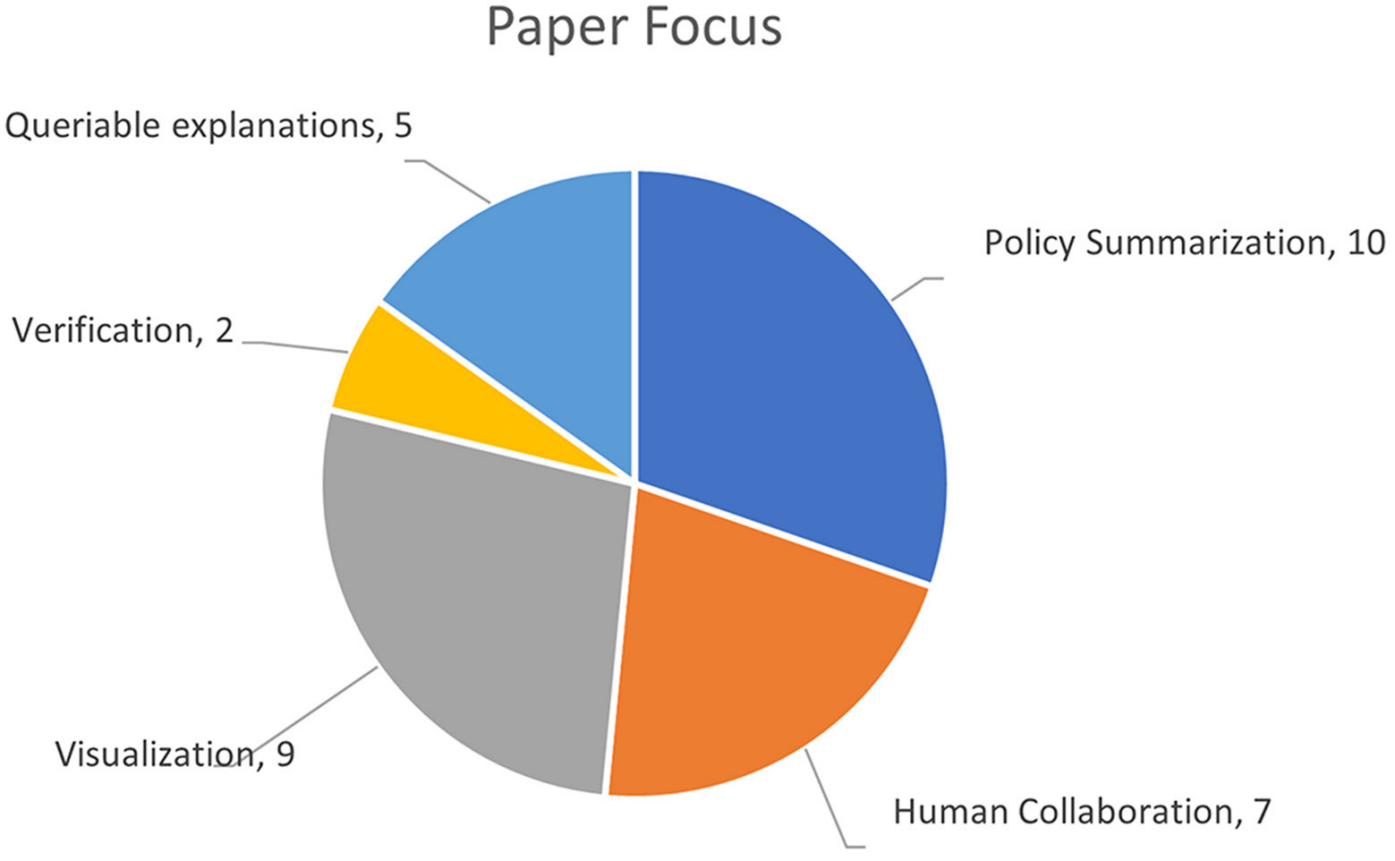
Categorization of papers by scope. Note that some papers were multi-faceted and covered multiple categories.

Table 1 summarizes which category was determined for each paper, and the distribution of papers across different domains is presented in Figure 5. These topics are used to help structure the following discussion section.

**Table 1:** A summary of the papers reviewed, categorized by purpose.

**Purpose**	**Papers**
Human collaboration	Amir et al. (2019), Hayes and Shah (2017), Huang et al. (2019), Pynadath et al. (2018), Tabrez et al. (2019), Tabrez and Hayes (2019), Ehsan et al. (2019)
Visualization	Dao et al. (2018), Dethise et al. (2019), Iyer et al. (2018), Joo and Kim (2019), Mishra et al. (2018), Pan et al. (2019), Wang et al. (2018), Greydanus et al. (2018), Yang et al. (2018).
Policy summarization	Amir et al. (2019), Fukuchi et al. (2017a,b), Hayes and Shah (2017), Lage et al. (2019), Madumal et al. (2020), Sridharan and Meadows (2019), Stamper and Moore (2019), Lyu et al. (2019), Verma et al. (2018)
Query-based explanations	Amir et al. (2019), Hayes and Shah (2017), Kazak et al. (2019), Sridharan and Meadows (2019)
Verification	Kazak et al. (2019), Dethise et al. (2019)

*Note that a paper could have multiple purposes*.

## Discussion

The following sections address each of the defined research questions for this work.

### RQ1: What Approaches Exist for Producing Explainable Output for Reinforcement Learning?

#### Human Collaboration

Seven papers discussed approaches that were inherently human-based in their approaches.

Pynadath et al. (2018) tested human interaction with an agent while manipulating the perceived ability of the agent by altering the explanations it gave. They explored the design of explanations for Partially Observable Markov Decision Process (POMDP)-based RL agents. The authors mapped different components of the POMDP model to the Situational Awareness-based Agent Transparency (SAT) model in order to determine a set of “explanation content” to assist with situational awareness in a military setting. The SAT was comprised of three levels:

- The agent's *actions and plans;*
- The agent's *reasoning process*, and;
- The agent's *predicated outcomes* (optionally including uncertainties).

The researchers were able to manipulate the ability of the agent in their experiments for human-machine team missions. They evaluated an explainable robot agent which would navigate around an online 3D environment. The robot used a scanner to recommend to the human team members what they should do next (enter the building, put on armor etc.) Example explanations for this agent included “I believe that there are no threats in the market square” for beliefs about the current state of the world, or “my image processing will fail to detect armed gunmen 30% of the time” as an explanation of the current state of the observation model the agent was using.

The authors evaluated differing levels of explanation and found that in general they could potentially “improve task performance, build transparency, and foster trust relationships.” Interestingly, the authors noted that explanations which resulted in users being uncertain about what to do next were considered just as ineffective as when no explanations were given. Ability of the robot was tested as well. The high-ability robot got predictions 100% correct, resulting in participants not questioning the robots' decisions (potentially leading those participants to ignore some of the explanation content as the robot “got it right anyway”). This is a prominent example of overreliance, mentioned earlier.

In a similar vein as the work by Pynadath et al. (2018) and Sridharan and Meadows (2019) contributed a theory of how to enable robots to provide explanatory descriptions of its decisions based upon its beliefs and experiences. Building upon existing work into scientific explanation, the theory encompassed 3 core components:

1. How to represent, reason with, and learn knowledge to support explanations.
2. How to characterize explanations in terms of axes of abstraction, specificity, and verbosity.
3. How to construct explanations.

The authors went on to describe an architecture which implemented this theory in a cross-domain manner. The architecture itself operates on two levels, first reasoning using commonsense domain knowledge at a high-level a plan of actions. The system utilized RL for the actions, working alongside Answer Set Prolog (ASP) reasoning of object constants, domain attributes, and axioms based upon state-action-reward combinations (Sridharan and Meadows, 2019). The ASP reasoning was used for planning and diagnostics, and to trigger the learning (using RL) of new concepts when something unknown is encountered (Sridharan and Meadows, 2018). When producing explanations, the architecture extracted words and phrases from a human query matching a template, and based upon human-controlled values effecting the abstraction, specificity, and verbosity of the explanation, reasoned based upon changes in beliefs about the environment. The two evaluation tasks used were moving objects to a target location and following a recipe.

Tabrez and Hayes (2019) described a framework called RARE (Reward Augmentation and Repair through Explanation) which also extended the POMDP model. Using this framework, the RL agent was able to infer based upon a human's behavior the most likely reward function they were using and communicate to the user important differences or missing information in the human's reward function. The agent autonomously provided “actionable statements,” which the authors tested in a controlled experiment on a Sudoku-style game. The control group were given an agent who would alert users who were about to make a mistake, and the treatment group had an agent which would indicate that a move would result in failure, and *explain* to them which rules of the game would be broken. Participants found the agent with explanations to be more helpful, useful, and intelligent. The authors however highlighted the fact that the approach does not scale. Statements used a template in the form of: “If you perform {describe action}, you will fail the task in state {describe state} because of {describe reward function difference}.”

Looking at autonomous vehicles as an example, Pan et al. (2019), contributed Semantic Predictive Control (SPC) which learns to “predict the visual semantics of future states and possible events based upon visual inputs and an inferred sequence of future actions” (p. 3203). Visual semantics in this case refers to object detection, and the authors suggested that these predicted semantics can provide a visual explanation of the RL process. The paper, however, provided little insight into how it addresses the problem of XAI.

Another work in the autonomous driving domain, Huang et al. (2019) compared approximate-inference and exact-inference approaches in an attempt to leverage the way humans make inferences about how a RL agent operates based upon examples of optimal behavior. Their work compared different approximate-inference models in a user study, where users were shown example behaviors. Users were tasked with selecting from a range of trajectories which one they thought the autonomous driver was most likely to take. The authors' findings suggested that an approximate-inference model using a Euclidean-based approach performed better than algorithmic teaching.

Finally, work by Ehsan et al. (2019) presented a novel approach for generating rationales (the authors note a distinction between this and explanations, indicating that rationales do not need to *explain* the inner workings of the underlying model). The method involves conducting a modified think-aloud user study of the target application (in this case, the game *Frogger*) where participants are prompted to verbally indicate their rationale for each action they take. These rationales (and the associated observation-action pairs in the game) are then cleansed and parsed before being fed through an encoder-decoder network to facilitate natural language generation of actions taken by a RL agent. The authors conducted user studies on the generated explanations compared to random and compared to pre-prepared human explanations. Generated explanations performed better than randomly generated explanations in all factors tested (confidence, human-likeness, adequate justification, and understandability), and performed similarly to the pre-prepared explanations, but did not beat it. A limitation of this work was that the system was designed for turn-based or distinct-step environments, and the authors are continuing their work to look at continuous environments. A major challenge in this is that data collection of rationales during the think-aloud stage is constrained to be after each action taken and would be an arduous process for a human for games larger than Frogger.

#### Visualization

Nine of the papers reviewed focused on graphical visualization of the agent learning process. Some remarkable visualizations have already been produced, however as discussed later, limitations exist in the ability of these visualizations to fully explain an agent's behavior or policy.

Wang et al. (2018) provided a comprehensive yet highly application-specific visualization tool for Deep-Q Reinforcement Learning Networks called *DQNViz*, with the goal of identifying and extracting typical action/movement/reward patterns of agents. While *DQNVis* was scoped to the Atari *Breakout* game and was focused primarily on objectives relating to improving the training of an agent during development, the tool shows the power of visualization techniques to gain insight into the behaviors of an agent.

**Figure 6:**
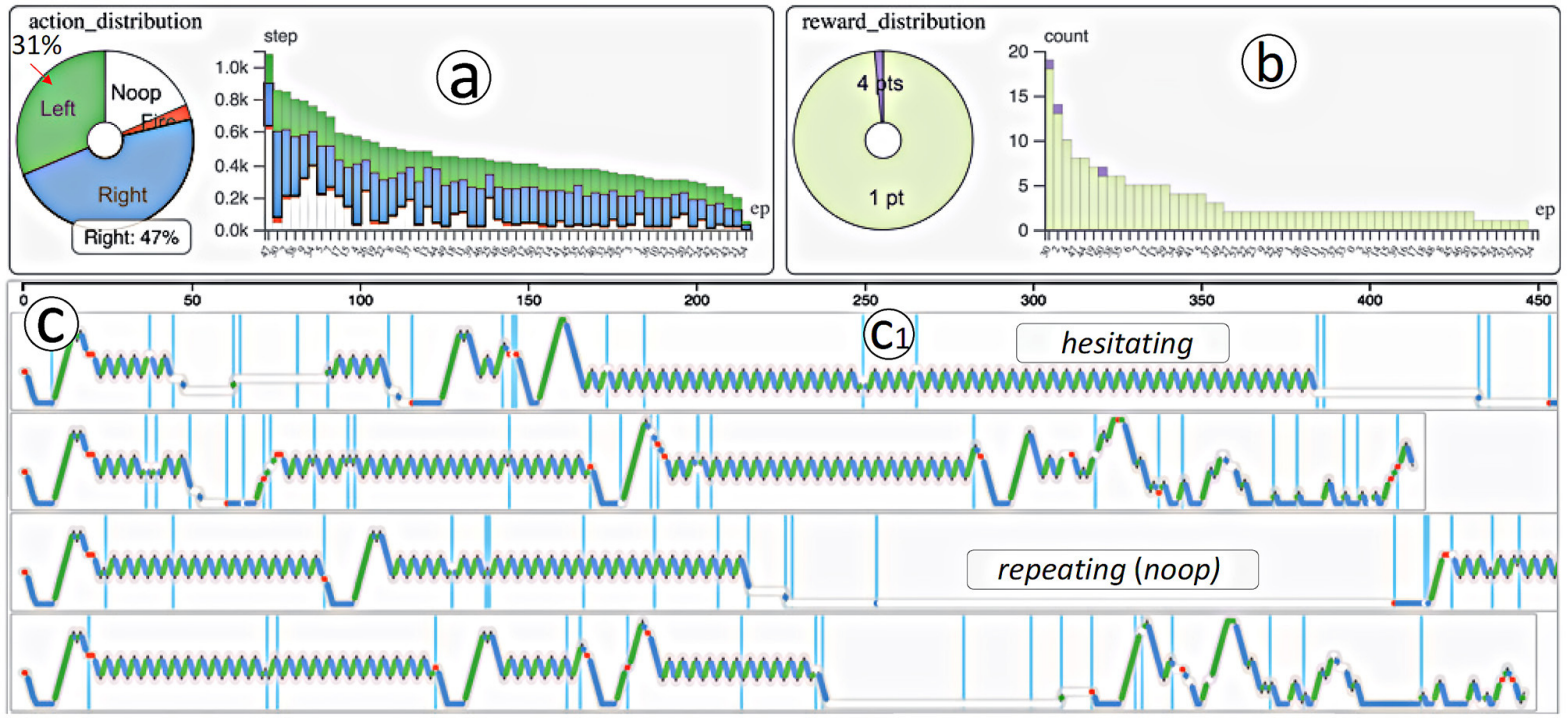
Example visualizations from DQNVis, showing **(a,b)** episode duration, and **(c)** actions taken over time and how experts identified these as “hesitating” and “repeating” behaviors which were non-optimal (from Wang et al., 2018, p. 294, reproduced with permission).

The system allowed behaviors to be identified and labeled using tools, such as regular expressions, principal component analysis, dynamic time warping, and hierarchical clustering. Core behaviors in *Breakout* that the agent went through during training included *repeating, hesitating, digging*, and *bouncing* (see Figure 6). The tool allowed users to investigate certain moments and see what the agent did at that time and highlight which states in each layer of the convolutional neural network were most activated. Coupled with video output surrounding certain behaviors, experts were able to explore what caused bad behaviors like repetition or hesitation.

Testing so far on DQNViz has been conducted only with deep learning experts who were involved in the initial collaborative process of building the system, and so the usability for non-experts remains to be seen.

Region Sensitive Rainbow (RS-Rainbow) was a visualization method contributed by Yang et al. (2018). RS-Rainbow used a “region-sensitive module” (p. 1) added in after the standard image convolution layers of a deep neural network, which looks for distinctive patterns or objects, and this representation replaces the original representation of the screen as the state used by the deep Q network agent. The authors provided three alternative approaches for visualizing the important regions: a weights-overlay, a soft saliency mask, and a binary saliency mask. Tested on a range of Atari games, the agent out-performed state-of-the-art approaches for Deep RL. The authors have not yet studied to what extent the visualization aids in human understanding in non-experts and ability to debug agents.

Greydanus et al. (2018) also presented a visualization technique tested on Atari games. They contributed *perturbation-based saliency*, to artificially reduce the RL agent's certainty about specific features (e.g., the location of the ball in the Atari game Pong), and its effect on the agent's policy. Using this, the authors could determine the regions of an image which had the most effect. The authors used the visualization to understand “strong” policies where agents perform dominant strategies (such as “tunneling” in *Breakout*), and to observe how attention changes while the agent learns. The study found that the visualization helped non-expert users with identify agents with overfitted models. Finally, the study showed how visualization can aid in debugging, showing examples of Atari games where human performance was not yet attained. In *MsPacman*, it was found that the agent was not tracking the ghosts, and in *Frostbite*, the agent was only tracking the player and goal, and not the destination platforms.

A similar approach to highlighting areas of an image that were relevant to a decision was presented by Joo and Kim (2019) who applied the Gradient-weighted Class Activation Mapping (*Grad-CAM*) approach, to Asynchronous Advantage Actor-Critic (A3C) deep RL in the context of Atari Games. The result was effectively a heatmap indicating which parts of the input image affected the predicted action.

**Figure 7:**
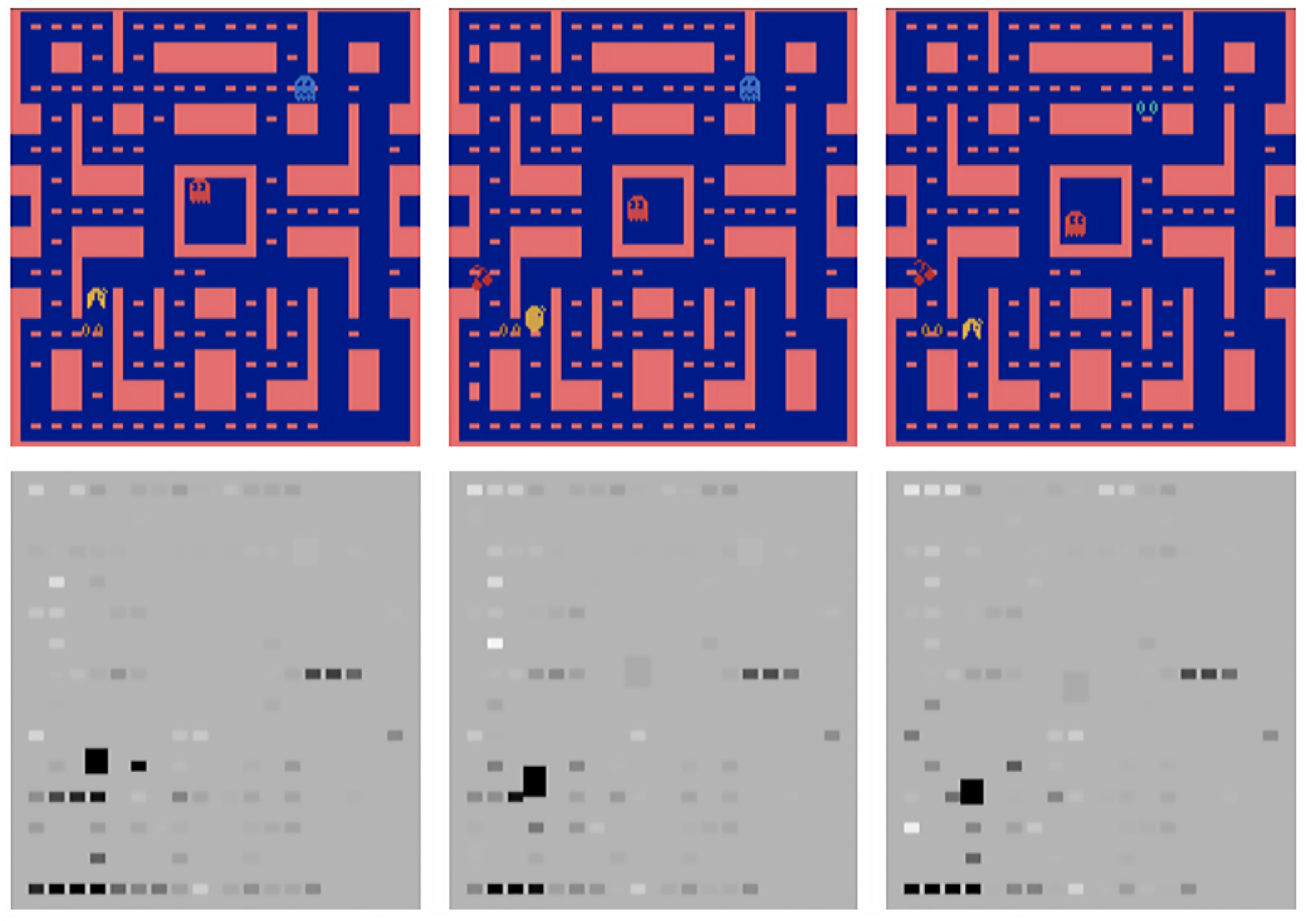
Screenshots (left) and their matching object saliency maps (right) in the game Ms Pacman (from Iyer et al., 2018, p. 148, reproduced with permission).

A more complex approach to visualizing the focus of a RL agent was presented by Iyer et al. (2018). The authors claimed their system could “automatically produce visualization[s] of their state and behavior that is intelligible to humans.” Developed within the domain of Atari games, the authors used template matching to detect objects in the screen input to produce a number of “object channels” (one for each detected object), as extra input into the convolutional neural network used by the RL agent. The authors also described an approach to produce a “pixel saliency map,” where pixels are ranked in terms of their contribution toward the chosen action in that state (see Figure 7). As the pixel map is generally not human intelligible (i.e., it is difficult to interpret due to noise and other factors), the approach was combined with the previously mentioned object detection, to produce an “object saliency map” which is easier for humans to understand. The authors tested the system using human experiments, where participants were tasked with generating explanations of the behavior of a Pacman agent, and predict the next action. Participants assisted by the object salience maps performed significantly better on the tasks.

Sparse Bayesian Reinforcement Learning (SBRL; Lee, 2017) can explain which relevant data samples influenced the agent's learning. An extension to SBRL by Mishra et al. (2018) was *V-SBRL* which was applied to vision-based RL agents. The system maintains snapshot storage to store important past experiences. The authors presented an approach to visualizing snapshots at various important locations (as determined by the SBRL algorithm), by showing state-action pairs. In the context of a navigation task, an interesting visualization was provided by overlaying the snapshots on a Q contour plot, allowing designers to see where the agent had confidence in its actions and where it did not. V-SBRL may prove to be useful in continuous environments, where the number of important moments may be high, but can be compressed down by finding similar state-action pairs within the continuous space. In another paper from the same authorship team, Dao et al. (2018) applied the approach to the Atari games Pong and Ms Pacman.

Pan et al. (2019) as previously described provided visual explanations in the form of object detection.

#### Policy Summarization

Ten papers provided approaches to in some way summarize the policy that a RL agent has learned. While a policy summary doesn't explain an individual action, it can help provide context for why an action was taken, and more broadly why an agent makes the overall set of actions it makes.

Fukuchi et al. (2017a) described the *Instruction-based Behavior Explanation* (IBE) approach which allows an agent to announce their future behavior. To accomplish this, the agent leveraged Interactive RL where experts provide instructions in real-time to beginner agents. The instructions are then re-used by the system to generate natural-language explanations. Further work by Fukuchi et al. (2017b) then expanded on this to a situation where an agent dynamically changed policy.

Hayes and Shah (2017) used code annotations to give human-readable labels to functions representing actions and variables representing state space, and then used a separate Markov Decision Process (MDP) to construct a model of the domain and policy of the control software itself. The approach is compatible not only with RL, but also with hard-coded conditional logic applications too.

The authors tested their approach on three different domains, a grid-world delivery task, the traditional Cart Pole task, and an inspection robot task. Generated policies were similar in nature to the expert-written policies. The authors suggested that the state space and action space of the learned domain model needs to be constrained in order for the approach to be effective, and to prevent combinatory explosion. It remains to be seen if the approach will work on environments more complex than the Cart Pole.

Amir et al. (2019) proposed a conceptual framework for strategy summarization. The framework consisted of three core components:

1. intelligent states extraction: which given the agent's strategy/policy as input, outputs a prioritized subset of states to be included in the summary—the main challenge being determining what the desirable characteristics of a state are for use in a summary;
2. world state representation: which involves the summarization of potentially complex world states (i.e., an agent may consider a large number of variables with different weights for certain decisions); and
3. the strategy summary interface: which is concerned with a usable and appropriate user interface for exploration of the summary, which is guided by both the user and the system itself.

For each of these components, the authors provided potential research directions for addressing these problems in the RL space, however this is the only paper reviewed which did not include an implementation which was tested alongside the theoretical framework.

Recent work by Madumal et al. (2020), implemented explanations in a RL agent playing *StarCraft II*, under the premise that humans would prefer causal models of explanation. The agent was able to answer “counterfactual” levels of explanations, i.e., “why” questions. The authors introduced an approach where a causal graph was generated in the form of a directed acyclic graph, where state variables and rewards were nodes, and actions being edges (assuming that an action *caused* a transition between different states). Using structural causal equations, on the causal graph, an explanation was generated.

The explainable agent was tested on 120 participants. To test participants understanding of the explanations, they were tasked with first watching the agent play StarCraft II and explain its actions, followed by watching an agent play and predict its next action. The agent was found to have statistically significantly higher levels of satisfaction and understanding of actions taken than a non-explainable agent. Interestingly however, no significant difference in levels of trust was found, a fact that the author attributed to the short interaction time with the agent.

**Figure 8:**
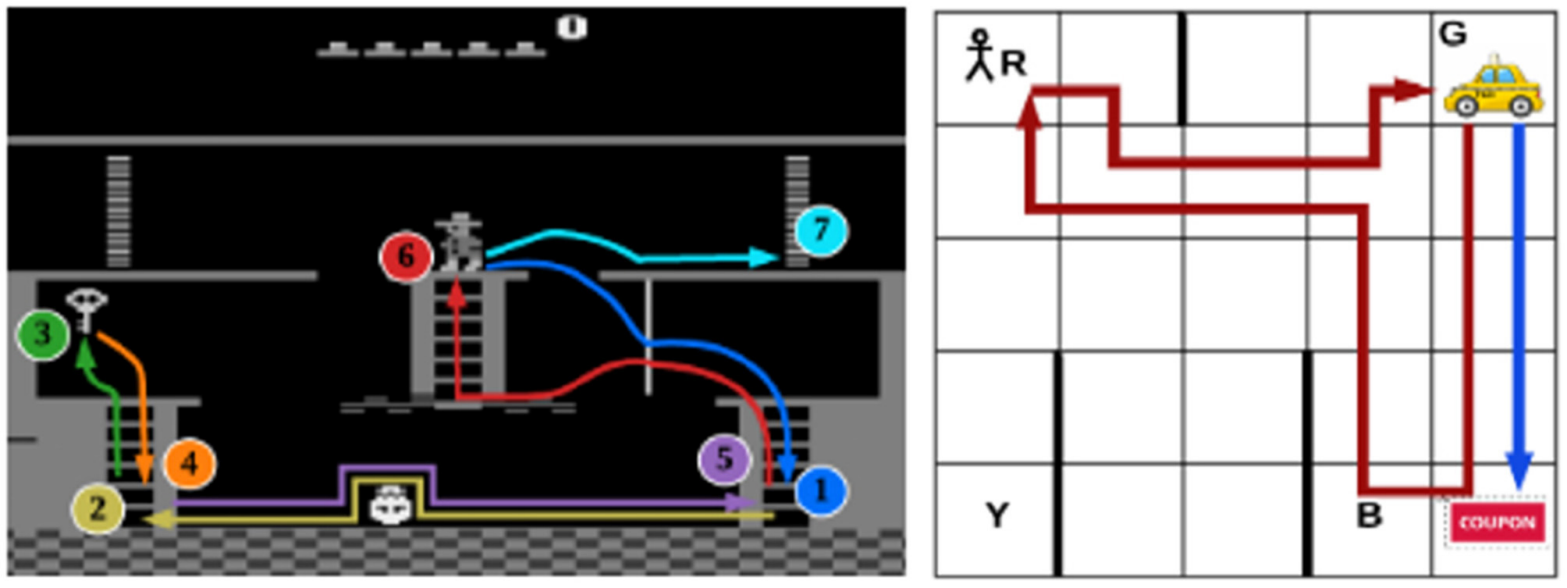
Summarized policies for Montezuma's Revenge (left), and the “taxi” problem (right), (from Lyu et al. (2019), p. 2975, reproduced with permission).

A set of causal rules was also used in similar work by Lyu et al. (2019) who proposed the Symbolic Deep Reinforcement Learning (SDRL) framework, aimed at handling high-dimensional sensory inputs. The system used symbolic planning as a high-level technique for structuring the learning with a symbolic representation provided by an expert. The high-level symbolic planner had the goal of maximizing some “intrinsic” reward of formulating the most optimal “plan” (where a plan is a series of learned sub-tasks). DRL was used at the “task/action” level to learn low-level control policies, operating to maximize what the authors call an “extrinsic” reward. The authors tested their new approach on the classic “taxi” Hierarchical Reinforcement Learning (HRL) task, and the Atari game *Montezuma's Revenge* (see Figure 8). While the system contributed gains in terms of data efficiency, of interest to this paper is the use of symbolic representation and the high-level planner. Such representation of the environment and action space and abstraction at a high-level can be useful in the pursuit of XAI as it may open up opportunities to (with careful design) provide more interpretable systems.

**Figure 9:**
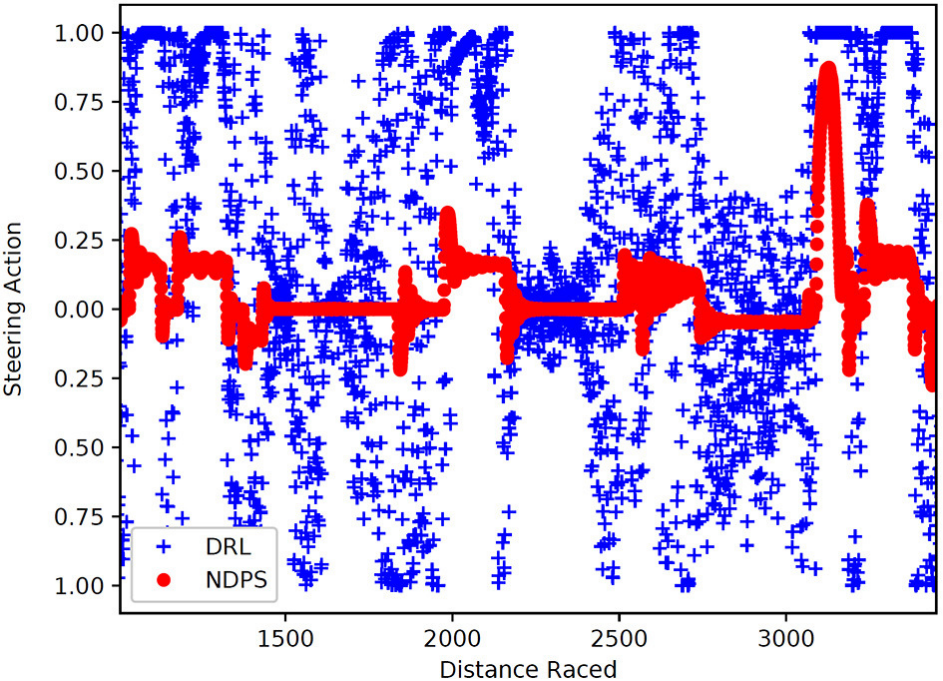
Plot of steering actions generated by standard DRL agent vs. the summarized NDPS policy, which resulted in much smoother steering movements (from Verma et al., 2018, p. 7, reproduced with permission).

Verma et al. (2018) described a framework for generating agent policies called Programmatically Interpretable Reinforcement Learning (PIRL), which used a high-level, domain-specific programming language, similar to the symbolic representations mentioned previously. The system used DRL for initial learning, and then a novel search algorithm called Neurally Directed Program Search (NDPS) to search over the DRL with a technique inspired by imitation learning to produce a model in the symbolic representation. The resulting model was described by the authors as “human readable source code” (p. 9), however no tests have yet been conducted on how well users can understand it, or how useful it is for debugging. The authors indicated that the resulting policy was smoother than the one generated by DRL—in the case of the test domain of a racing game, the steering output was much smoother, albeit with slower lap times (see Figure 9).

Lage et al. (2019) reported on different approaches for agent policy summarization, using Inverse Reinforcement Learning and Imitation learning approaches. Tested in three different domains, the authors found that the policy of an agent was most accurately reproduced when using the same model that was used for extraction as was used for reconstruction. Stamper and Moore (2019) compared policies generated by machines to those of humans. Using *post-hoc* human inspection, they analyzed data from a DQN RL agent, using t-SNE embedding. They found that the agent playing *Space Invaders* exhibited similar decision-making behaviors to expert human players (e.g., keeping the invaders in a square formation, and destroying right-most enemies first to slow down the rate of advancement). The work is still in its early stages, and the authors plan to automate the strategy identification process.

The previously described work by Sridharan and Meadows (2019) also provided for a summary of learned policy in their approach at different levels of abstraction. These summaries were able to be queried by the user, as explained in the next section.

#### Query-Based Explanations

Five papers described an interactive query-based approach to extracting explanations from a RL agent. Hayes and Shah (2017) went into the most detail. Broadly, their system conducted 4 core actions:

1. identify the question based upon a template approach, e.g., “When do you do {action}?”;
2. resolve states [using the template from (1), determine the states that are relevant to the question];
3. summarize attributes (determine common attributes across the resolved states); and
4. compose a summary in a natural language form (using “communicable predicates”).

These steps integrated with the code annotations previously described for this system.

The work by Madumal et al. (2020) featured a query-based RL agent playing *Starcraft II*. The agent focused on handling *why?* and *why not?* questions. An example question was provided by the author, “Why not build barracks?”, to which the agent replied, “Because it is more desirable to do action build_supply_depot to have more supply depots as the goal is to have more destroyed units and destroyed buildings.” This is a great example of a RL agent being able to answer questions about its action, however it remains to be seen how well this approach will scale.

Kazak et al. (2019) presented an approach which allowed experts to query a RL agent in order to perform verification tasks. In their tests, queries took over 40 s to complete. Their work is described in more detail in the verification section, as that was the primary purpose of that work.

Previously described work on policy summarization by Amir et al. (2019) and Sridharan and Meadows (2019), both highlighted the importance of being able to further query summarized policies in order to prevent initial cognitive load on the user by presenting a policy that was too complex or verbose. The query functionality in Sridharan and Meadows (2019) was able to be customized to different levels of abstraction, specificity, and verbosity, but this was further guided by the ASP-based architecture they used.

#### Verification

A theme which was found within two reviewed papers was that of verification. Verification is an area of importance to RL for a number of reasons, not least due to the impact on safety it can have. As Fulton and Platzer (2018) point out, formal verification allows us to detect discrepancies between models and the physical system being controlled, which could lead to accidents.

Acknowledging the non-explainability of RL systems, Kazak et al. (2019) suggested that verifying that systems adhere to specific *behaviors* may be a good alternative to verifying that they adhere to exact values from a model. They presented an approach called *Verily*, which checks that the examined system satisfies the pre-defined requirements for that system by examining all possible states the agent could be in, and using the formal verification approach *Marabou*. The system identifies “undesirable” sequences using bounded model checking queries of the state space. Of interest to this review is that when a system is found to not meet the requirements, a *counter example* is generated that *explains* a scenario in which the system fails. The authors tested this approach on three case studies within a networking/cloud computing domain, providing verification that the RL systems employed were conducting desired behaviors and avoiding poor outcomes (e.g., verifying that an adaptive video streaming system was correctly choosing between high- or low-quality video based upon network conditions). The impact of *Verily* on the trust relationship between humans and the systems remains to be tested, as does the scalability of this approach since it operates on all possible states.

Similar to the Kazak et al. study was work by Dethise et al. (2019), also in the domain of RL for networking problems. They looked at using interpretability tools to identify unwanted and anomalous behaviors in trained models. The system in question was *Pensieve*, an adaptive bit rate selector, common in video streaming services. The authors analyzed the relationship between data throughput and decisions made by the agent. Using simple visualization techniques, they showed that the agent never chose certain bandwidths for users (despite there being no bias present in the training data). Further analysis revealed that the agent preferred to multiplex between higher and lower bitrates when the average available bitrate was one of the identified ignored bitrates. The authors also analyzed which features contributed the most to decisions, finding that the most highly weighted feature was the previous bit rate. This paper used domain knowledge to lead a guided exploration of the inputs of a relatively simple RL agent, however some of the approaches and visualizations presented may be of use in other areas.

### RQ2: What Are the Limitations of Studies in the Area of XAI for Reinforcement Learning?

In reviewing the collected papers, a number of common limitations were identified, particularly in the use of “toy examples,” a lack of new algorithms, lack of user testing, complexity of explanations, basic visualizations, and lack of open-sourced code. The following sections discuss in more detail the various common limitations.

#### Use of Toy Examples, Specific Applications, and Limited Scalability

Given the early stages of XAI for RL research, all papers reviewed presented effectively “toy” examples, or case studies which were deliberately scoped to smaller examples. In most cases this was done to avoid the combinatory explosion problem in which where state- and action-space grow, so do the number of possible combinations of states and actions. An example of this was Hayes and Shah (2017) who scoped their work to the basic Cart Pole environment. Similarly the Tabrez and Hayes (2019) paper focused on a grid-world example.

Many authors indicated limitations in scaling the approach to more complex domains or explanations (with the exception of Sridharan and Meadows, 2019, who indicated that a strong contribution of their work was that their approach would scale). Sixteen of the papers reviewed were either agents within video games or were tested with video game problems, and surprisingly few were on more real-world applications such as autonomous driving or robotics. Examples of this include Ehsan et al. (2019) who provide an interesting example but is highly scoped to the *Frogger* game, and Madumal et al. (2020) who looked at Starcraft II. While this is naturally following on from the success of DeepMind, and video game problems provide for challenging RL tasks, there is an opportunity for more work on applications outside of this domain.

#### Focus on Modification of Existing Algorithms

Papers examined in this review described RL approaches or visualization techniques to augment or accompany existing RL algorithms. There is an opportunity in this area to design RL algorithms with explainability in mind. Symbolic representation can be a step toward allowing for inherently explainable and verifiable agents.

#### Lack of User Testing

A major limitation of the studies presented in this review is that many approaches were either not tested with users (17 papers), or when they did, limited details of the testing were published, failing to describe where the participants were recruited from, how many were recruited, or if the participants were knowledgeable in Machine Learning (Pynadath et al., 2018; Tabrez and Hayes, 2019; Tabrez et al., 2019). Participant counts varied greatly, with one paper using 3 experts (Wang et al., 2018), others with students (Iyer et al., 2018), *n* = 40; and Greydanus et al. (2018), *n* = 31, and three recruiting using Amazon Mechanical Turk^3^ (Huang et al., 2019, *n* = 191; Madumal et al., 2020, *n* = 120; and Ehsan et al., 2019, *n* = 65 and *n* = 60).

This lack of user testing across the reviewed papers is consistent with the findings in the Miller et al. (2017) review of XAI in Machine Learning.

#### Explanation Presentation

In some cases, implementations provided too much information for the human participant, or required significant additional knowledge from the human team member, making these approaches unsuitable for use by laypeople or even knowledgeable domain experts. This finding is consistent with the survey paper by Miller et al. (2017) who found that there is very little research in the XAI space on leverages existing work on how people “generate, select, present, and evaluate” (p. 4) explanations, such as the work by Lombrozo (2007) which describes how people prefer simpler and more general explanations over specific and more likely explanations.

In the papers focusing on visualization, most expanded on existing techniques of pixel saliency which have successfully been used for image classification (e.g., Greydanus et al., 2018; Iyer et al., 2018; Yang et al., 2018). RL problems happening over time may need more complex visualization techniques to capture the temporal dimension. Other forms of visualization presented were primarily 2D graphs (e.g., DQNVis, Wang et al., 2018), however these solutions may struggle to scale and to be interpretable in more complex domains given the large amount of data involved network design.

The majority of papers with user studies presented explanations or visualizations palatable only to experts. Further research could look at providing explainable systems targeted at laypeople or people more likely to be working with the agent, rather than those with a background in artificial intelligence. Symbolic representation was present in a number of papers in this review (e.g., Verma et al., 2018; Lyu et al., 2019). Future research could consider alternatives to text representation of these to provide more visceral explanations, such as annotations in the virtual environment. Similarly, visualization techniques presented in the papers in this review are a good start (e.g., DQNVis, Wang et al., 2018), however the toolkits provided may be enhanced by the addition of visualization techniques better designed for handling the temporal dimension of RL (such as the Immersive Analytics Toolkit by Cordeil et al. (2019) or TensorBoard graphs^4^), as well as multi-modal, immersive forms of visualization such as virtual or augmented reality to better explore the complex data structures of neural networks (Marriott et al., 2018).

#### Lack of Open-Source Code

Finally, only four papers provided the reader with a link to the open-source repository of their code (Greydanus et al., 2018; Yang et al., 2018; Dethise et al., 2019; Sridharan and Meadows, 2019). This lack of availability of code could be as the result of many things, but we argue that given the toy example nature of the work previously described, that some authors didn't find utility in providing code online. Additionally, intellectual property issues can sometimes arise, making it not possible to publish code in an open-source matter. This is despite the potential benefits for the academic community of shared, open-source code.

## Conclusion

The area of XAI is of growing importance as Machine Learning techniques become commonplace, and there are important issues surrounding ethics, trust, transparency, and safety to be considered. This review has explored the extant literature on XAI within the scope of RL. We have shown that work in this area is still in its early stages but growing in prevalence and impact it can make. Clear trends are appearing in terms within the area with researchers focusing on human collaboration, visualization techniques, whole-of-policy summarization explanations, query-based explanations, and verification approaches.

This paper has described current approaches, while also identifying a range of limitations in this field of research, primarily finding a lack of detail when describing human experiments, limited outcomes in terms of scalability and level of comprehension of explanations for non-expert users, and under-use of more advanced visualization techniques such as multi-modal displays and immersive visualization. To truly break through the black box of RL, a strong combination of well-articulated explanations coupled with advanced visualization techniques will be essential tools for Machine Learning experts and users alike.

## Data Availability Statement

The raw data supporting the conclusions of this article will be made available by the authors, without undue reservation.

## Author Contributions

LW completed the majority of the work here, under the supervision of TB. TB contributed major edits and comments which helped shape the overall project. All authors contributed to the article and approved the submitted version.

## Conflict of Interest

The authors declare that the research was conducted in the absence of any commercial or financial relationships that could be construed as a potential conflict of interest.

## Footnotes

^1^Available online at: https://cloud.google.com/explainable-ai.

^2^Available online at: https://arxiv.org/.

^3^Available online at: https://www.mturk.com/.

^4^Available online at: https://www.tensorflow.org/tensorboard/graphs.
