# Supplementary material for: Explainable AI and Reinforcement Learning—A Systematic Review of Current Approaches and Trends
Source: Front Artif Intell. 2021 May 20;4:550030. doi: 10.3389/frai.2021.550030 (PMC8172805; doi:10.3389/frai.2021.550030)
Supplement: Supplementary file 1 [file Data_Sheet_1.docx]

Supplementary Material

# Full Boolean Search Queries

Below is the complete set of search strings used. Searches were conducted on the ACM, IEEExplorer, Science Direct, and Springer Link digital libraries.

- ("data visualization" OR "information visualization" OR "knowledge visualization") AND ("reinforcement learning")
- ("data visualization" OR "information visualization" OR "knowledge visualization") AND ("reinforcement learning") AND ("explanation")
- ("data visualization" OR "information visualization" OR "knowledge visualization") AND ("reinforcement learning") AND ("explainable ai")
- ("data visualization" OR "information visualization" OR "knowledge visualization") AND ("reinforcement learning") AND ("XAI")
- ("data visualization" OR "information visualization" OR "knowledge visualization") AND ("reinforcement learning") AND ("black box")
- ("reinforcement learning") AND ("explainable")
- ("reinforcement learning") AND ("explanation")
- ("reinforcement learning") AND ("explainable ai")
- ("reinforcement learning") AND ("XAI")
- ("reinforcement learning") AND ("visual analytics")
- ("reinforcement learning") AND ("hybrid analytics")
- ("reinforcement learning") AND ("human-in-the-loop")

# Summary Table of Results

| **Author** | **Title** | **Purpose** | **Theory or Method** | **Domain / Example** | **Publication Type** | **User Study (n)** |
| --- | --- | --- | --- | --- | --- | --- |
| Amir et al. (2019) | Summarizing agent strategies. | Policy Summarization  Human Collaboration  Query-based explanations | Theory | Robotics | Journal | - |
| Dao et al. (2018) | Deep Reinforcement Learning Monitor for Snapshot Recording | Visualization | Method | Games  Gridworld  Pong  Pacman | Conference | - |
| Dethise et al. (2019) | Cracking Open the Black Box: What Observations Can Tell Us About Reinforcement Learning Agents | Visualization  Verification | Method | Networking  Video bitrate | Conference | - |
| Fukuchi et al. (2017a) | Application of Instruction-Based Behavior Explanation to a Reinforcement Learning Agent with Changing Policy | Policy Summarization | Method | Games | Conference | - |
| Fukuchi et al. (2017b) | Autonomous Self-Explanation of Behavior for Interactive Reinforcement Learning Agents | Policy Summarization | Method | Games | Conference | - |
| Hayes and Shah (2017) | Improving Robot Controller Transparency Through Autonomous Policy Explanation | Policy Summarization  Human Collaboration  Query-based explanations | Method | Robotics  CartPole  Gridworld | Conference | - |
| Huang et al. (2019) | Enabling robots to communicate their objectives. | Human Collaboration | Theory | Autonomous Vehicles | Journal | Mechanical Turk (*n* = 191) |
| Iyer et al. (2018) | Transparency and Explanation in Deep Reinforcement Learning Neural Networks | Visualization | Method | Games  Pacman | Conference | University Students (*n*= 40) |
| Joo and Kim (2019) | Visualization of Deep Reinforcement Learning using Grad-CAM: How AI Plays Atari Games? | Visualization | Method | Games | Conference | - |
| Kazak et al. (2019) | Verifying Deep-RL-Driven Systems | Verification  Query-based explanations | Method | Networking  Video bitrate | Conference | - |
| Lage et al. (2019) | Toward Robust Policy Summarization | Policy Summarization | Theory | Games  Pacman | Conference | - |
| Madumal et al. (2019) | Explainable reinforcement learning through a causal lens | Policy Summarization  Query-based explanations | Theory | Games  Starcraft II | arXiv | Mechanical Turk (*n*= 120) |
| Mishra et al. (2018) | Visual Sparse Bayesian Reinforcement Learning: A Framework for Interpreting What an Agent Has Learned | Visualization | Method | Gridworld | Conference | - |
| Pan et al. (2019) | Semantic Predictive Control for Explainable and Efficient Policy Learning | Visualization | Method | Autonomous Vehicles  Games  GTA V  Flappy Bird | Conference | - |
| Pynadath et al. (2018). | Transparency Communication for Machine Learning in Human-Automation Interaction | Human Collaboration | Theory | Defence / Military | Book Chapter | Yes  (no details) |
| Sridharan and Meadows (2019) | Towards a Theory of Explanations for Human–Robot Collaboration | Policy Summarization | Theory | Robotics | Journal | - |
|  |  | Query-based explanations |  |  |  |  |
| Stamper and Moore (2019) | Exploring Teachable Humans and Teachable Agents | Policy Summarization | Method | Games  Space Invaders  Connect 4 | Conference | - |
| Tabrez et al. (2019) | Explanation-Based Reward Coaching to Improve Human Performance via Reinforcement Learning | Human Collaboration | Method | Robotics  Games  Soduku | Conference | Yes (no details) |
| Tabrez and Hayes (2019) | Improving Human-Robot Interaction Through Explainable Reinforcement Learning | Human Collaboration | Method | Gridworld | Conference | Yes  (no details) |
| Wang et al. (2019) | DQNViz: A Visual Analytics Approach to Understand Deep Q-Networks. | Visualization | Method | Games  Breakout | Conference | Experts  (*n* = 3) |
| Ehsan et al. (2019) * | Automated rationale generation: a technique for explainable AI and its effects on human perceptions | Human Collaboration | Method | Games  Frogger | Conference | Turk Prime  (*n* = 60; *n* = 65) |
| Greydanus et al. (2017) * | Visualizing and understanding atari agents | Visualization | Method | Games  Breakout  Ms Pacman  Frostbite  Enduro | arXiv | University Students (*n* = 31) |
| Lyu, et al. (2019) * | Sdrl: Interpretable and data-efficient deep reinforcement learning leveraging symbolic planning | Policy Summarization | Method | Taxi Domain  Gridworld  Games  Montezuma's Revenge | Conference | - |
| Verma et al. (2018) * | Programmatically interpretable reinforcement learning. | Policy Summarization | Method | Games  Torcs Car Racing | arXiv | - |
| Yang et al. (2018) * | Learn to interpret atari agents. | Visualization | Method | Games  Beam Rider  Enduro  Frostbite  Ms Pacman  Pong  Space Invaders | arXiv | - |

* indicates paper found through snowball sampling method
